# Effectiveness of cognitive behavioural therapy (CBT) interventions for anxiety in patients with chronic obstructive pulmonary disease (COPD) undertaken by respiratory nurses: the COPD CBT CARE study: (ISRCTN55206395)

**DOI:** 10.1186/1471-2466-13-62

**Published:** 2013-11-04

**Authors:** Karen Heslop, Julia Newton, Christine Baker, Graham Burns, Debbie Carrick-Sen, Anthony De Soyza

**Affiliations:** 1 Chest Clinic, The Newcastle upon Tyne Hospitals NHS Foundation Trust, Newcastle, Tyne & Wear NE1 4LP, UK; 2 Institute of Cellular Medicine, Newcastle University, Newcastle upon Tyne, Tyne & Wear NE1 7RU, UK; 3 NIHR Biomedical Research Centre in Ageing and Chronic Disease, Newcastle, UK; 4 Psychology Department, The Newcastle upon Tyne Hospitals NHS Foundation Trust, Newcastle upon Tyne, Tyne & Wear NE1 4LP, UK

**Keywords:** Anxiety, Chronic obstructive pulmonary disease (COPD), Cognitive behavioural therapy (CBT), Depression, Respiratory nurses

## Abstract

**Background:**

Anxiety and depression are common co-morbidities in patients with chronic obstructive pulmonary disease (COPD). Serious implications can result from psychological difficulties in COPD including reduced survival, lower quality of life, and reduced physical and social functioning, increased use of health care resources and are associated with unhealthy behaviours such as smoking. Cognitive behavioural therapy (CBT) is a psychological intervention which is recommended for the treatment of many mental health problems including anxiety and depression. Unfortunately access to trained CBT therapists is limited. The aim of this study is to test the hypothesis that CBT delivered by respiratory nurses is effective in the COPD population. In this paper the design of the Newcastle Chronic Obstructive Pulmonary Disease Cognitive Behavioural Therapy Study (Newcastle COPD CBT Care Study) is described.

**Methods/Design:**

This is a prospective open randomised controlled trial comparing CBT with self-help leaflets. The primary outcome measure is the Hospital Anxiety & Depression Scale (HADS) – anxiety subscale. Secondary outcome measures include disease specific quality of life COPD Assessment Tool (CAT), generic quality of life (EQ5D) and HADS-depression subscale. Patients will be followed up at three, six and 12 months following randomisation.

**Discussion:**

This is the first randomised controlled trial to evaluate the use of cognitive behavioural therapy undertaken by respiratory nurses. Recruitment has commenced and should be complete by February 2014.

**Trial registration:**

Current Controlled Trials, ISRCTN55206395

## Background

COPD is an umbrella term used to describe chronic bronchitis and emphysema which cause irreversible obstruction of the airways. COPD is increasingly felt to be a systemic syndrome with multiple co morbidities. Anxiety and depression are amongst the most common co-morbidities in COPD. Cognitive behavioural therapy (CBT) is a psychological intervention which is recommended for the treatment of many mental health problems including anxiety and depression. A breathing test called spirometry measures how much air can be exhaled in 1 second (FEV_1_) and the forced vital capacity (how much air can be exhaled fully after a full inhalation). The severity of obstruction is defined as, mild (FEV_1_ > 80% predicted), moderate (FEV_1_ 50-79%), severe (FEV_1_ 30-49%) and very severe [1]. The main cause of COPD is smoking [2].

Symptoms of COPD are cough, sputum production, wheeze and breathlessness. Symptoms of COPD lead to a gradual progression of disability over many years. As a consequence day to day functioning is affected and quality of life is reduced [3,4]. Patients focus on feeling unwell, their inability to perform everyday activities, and on the emotional consequences of the disease [5]. People with COPD are two to three times more likely to experience mental health problems than the general population [6]. Serious implications for people with COPD and mental health problems include poorer clinical outcomes, lower quality of life, reduced ability to manage physical symptoms effectively and are associated with unhealthy behaviours such as smoking [6] and reduced survival [3].

A systematic review and meta-analysis reported the prevalence of clinically significant anxiety at 36% and 40% for depression in patients with COPD [7]. Anxiety is an unpleasant emotional state associated with fear and distressing physical symptoms including rapid breathing and shortness of breath [8]. These symptoms overlap with the symptoms of COPD [9]. Anxiety is a significant predictor of the frequency of hospital admissions and re-admissions for acute exacerbations of COPD [10].

Panic disorder is up to ten times more prevalent in patients with COPD than in the general population [11,12] Panic disorder consists of recurring, unforeseen panic attacks. This is followed by persistent worry about having further attacks. Panic attacks develop suddenly, are associated with intense fear, anxiety, and physical arousal and are relatively short lived [8]. In patients with COPD, worsening breathlessness is often interpreted in a catastrophic way. Patients commonly think they cannot breathe and death is imminent. Symptoms of increased physical arousal follow leading to an escalating cycle which results in panic [13]. Patients become anxious about becoming breathless and avoid exertion which may trigger unpleasant symptoms occurring. This leads to physical de-conditioning thereby compounding exertional breathlessness, reducing confidence, which collectively exacerbates the panic cycle.

Management of psychological problems in COPD remains poor [14]. Guidelines for the management of anxiety and depression [15,16] recommend psychological treatment, pharmacological treatment or both in combination. Psychological interventions include CBT, counselling and self-help approaches. There are a growing number of studies evaluating the efficacy of psychological interventions to improve the psychological well-being of patients with COPD. Most of the published studies have used cognitive behavioural therapy. There is some evidence in a systematic review that psychological interventions such as cognitive behavioural therapy impact on anxiety [14]. However, a meta-analysis revealed that most studies were under-powered and demonstrated a small effect for anxiety only [14]. There is a need for high-quality systematic research of CBT in the routine management of patients with COPD.

A brief cognitive behavioural treatment intervention has been developed specifically for patients with COPD. The Lung Manual Treatment Program is based on principles of CBT and self-management [17]. The intervention is undertaken by respiratory nurses and aims to reduce anxiety, depression, improve quality of life and have health economic benefits. A randomised controlled trial has been developed to evaluate the efficacy of a brief CBT intervention undertaken by respiratory nurses in this patient group. The design and methods of the study are now presented.

## Methods/Design

### Design

The Newcastle COPD CBT CARE study is a prospective two armed randomised controlled trial (RCT) with longitudinal follow up of 12 months. The main aim of the trial is to identify if respiratory nurse delivered CBT, reduces symptoms of anxiety in patients with COPD. Data will be collected three, six and 12 months following randomization. Data collected at each time point can be found in Table 1.

**Table 1:** Outcome measures and time of assessment in Newcastle COPD CBT CARE study

	**Baseline**	**Follow up visit 1– 12–14 weeks**	**Follow up visit 2–6 months**	**Follow up visit 3–12 months**
Demographic Data	√	-	-	-
Gender	√	-	-	-
Age	√	-	-	-
Smoking Status & pack years	√	-	-	√
Ethnic Group	√	-	-	-
Marital Status	√	-	-	√
Education	√	-	-	-
Inhaler Technique	√	-	-	-
HAD - A	√	√	√	√
HAD - D	√	√	√	√
CAT	√	√	√	√
EQ5D	√	√	√	√
Spirometry	√	-	-	-
Oxygen saturations	√	-	-	-
MRC Dyspnoea Score	√	-	-	-
Co-morbidities	√	√	√	√
BMI	√	√	√	√
Medication	√	√	√	√
Health care utilisation (primary & secondary care)	√	-	-	√
Participation in pulmonary rehabilitation	√	√	√	√

√ refers to data collected at each visit.

### Primary outcome

The primary objective is to investigate if CBT delivered by respiratory nurses with either advanced CBT training (Post Graduate Diploma) or basic training in CBT techniques, reduces anxiety. The HADS Hospital Anxiety & Depression Scale (HADS) will be used to measure anxiety. Patients will be followed up at three, six and twelve months following randomisation into the study. The effects of the CBT intervention will be compared to usual care. A standardised manualised brief CBT intervention will be used. 224 patients will be required to complete the primary outcome at three months (112 in each arm). Patients will be stratified according to severity of COPD and age at randomisation.

### Secondary outcomes

Six questions have been developed as secondary outcomes. Firstly, does CBT delivered by respiratory nurses reduce hospital admissions in the 12 months following randomisation? Secondly, does CBT delivered by respiratory nurses reduce anxiety at six and 12 months compared to standard care? Thirdly, identify if CBT more effectively delivered by basic (three day short course) CBT trained respiratory nurses or advanced (postgraduate diploma in CBT) CBT trained respiratory nurses? Fourth, if CBT delivered by respiratory nurses reduce depression as measured by Hospital Anxiety and Depression Scale – Depression (HADS-D) at three, six and 12 months compared to standard care? Fifth, does CBT delivered by respiratory nurses improve patient quality of life at three, six & 12 months as measured by a reduction in COPD Assessment Tool (CAT) and EuroQol 5 Dimension questionnaire (EQ5D) scores? Finally, can this study inform design of a multicentre RCT?

### Setting

The setting for this study is an urban population in the North East of England. Patients who attend the respiratory clinics at Newcastle upon Tyne Hospitals NHS Foundation Trust with a diagnosis of COPD are routinely asked to complete a HADS questionnaire. All patients with a confirmed diagnosis of COPD with an FEV1/FVC ratio <0.7 who have a HADS score of ≥8 are offered information about the study. The unit already runs a respiratory nurse led CBT service, supervised and supported by a Consultant Clinical Psychologist (CB). Patients are offered between two and six individualised CBT appointments.

### Research governance

Approvals for this study were obtained from Sunderland Regional Ethics Committee. The trial has been entered onto the U.K. NIHR Clinical Research Network (CRN) Portfolio (Reference UKCRN Study ID: 10519; http://public.ukcrn.org.uk/search/StudyDetail.aspx?StudyID=10519 and ISRCTN study 55206395).

### Inclusion criteria

• Patient with a confirmed diagnosis of COPD (FEV1/FVC ratio <70% [5].

• All levels of COPD disease severity will be eligible including mild to moderate (FEV1 >50% predicted) and severe to very severe (FEV1 <50% predicted).

• Patients with HADS –anxiety subscale scores of ≥8.

• Willing to participate in the study and provide written consent.

• Agree to attend a minimum of two and maximum of six CBT sessions.

### Exclusion criteria

• Patients with a HADS-A score <8.

• Patients with known psychiatric history such as psychosis.

• Patients currently receiving psychological talking therapy including CBT treatment.

• Patients with cognitive impairment (e.g. dementia).

• Patients involved in any other interventional clinical trial.

### Randomisation

Patients enrolled in the study are randomised on a 1:1: basis to CBT or control using a computer generated software package after signing the consent form. Patients are stratified at randomisation for COPD disease severity using NICE 2010 categories (mild to moderate and severe to very severe) and age. Patient initials, date of birth and severity of COPD are entered into the web-based system, which returns the allocation status. Patients are informed of their treatment group following randomisation.

### Standard care

Both groups receive standard medical care which involves recording spirometry, oxygen saturation levels and HADS questionnaire at each routine medical clinic visit. Patients randomised to standard care arm of this study are provided with written information on anxiety and/or depression (Northumberland, Tyne & Wear Mental Health Trust leaflets). Patients are advised to contact their primary care team should further help be required. Treatment in primary care will be directed by primary care team and is not part of the trial intervention. It may include psychotherapy, counselling, antidepressants and/or anxiolytics. Information on primary care directed interventions will be recorded at each study follow up visit.

### CBT intervention

The study intervention involves standard care plus a brief CBT based program delivered by respiratory nurses. Clear manualised treatment will be used to ensure the CBT based treatment can be replicated. A manualised protocol has been specifically developed for this study by the Respiratory Nurse (KH) & Consultant Psychologist (CB). The CBT will be delivered by four respiratory nurses (two trained to Post Graduate Diploma Level and two who have completed a three day training course at foundation level). Monthly clinical supervision will be provided for all four nurses by a Consultant Clinical Psychologist (CB). The intervention will be delivered to individual patients in either in a respiratory clinic in secondary care or within the patients’ home. The CBT intervention is tailored to the patient’s individual needs (within the boundaries of the manual). Patients will be offered between two – six sessions of therapy as required. The number of sessions will be based on the patient’s progress and HADS scores. The first session will take 30–60 minutes. Subsequent follow up sessions will take up to 30 minutes.

The intervention includes the following processes. The nurse assesses the patient’s reported current difficulties and develops an individualised treatment plan. The treatment plan may include the development of coping strategies including goal-setting, identifying, challenging and changing negative thoughts, distraction, breathing control, problem-solving, activity scheduling/diary, relaxation, weighing up pros and cons; positive logs, learning to respond appropriately to symptoms and reducing avoidance and safety behaviours that maintain anxiety and low mood.

### Quality control of manualised CBT program

Quality control will be maintained through adherence to the study protocol, the principles of good clinical practice, research governance and clinical trial regulations. Data is being collected to assess consistency of intervention delivered in the treatment arm across nurses and principles of delivery of CBT. Sixty (5%) of the CBT sessions will be video recorded to validate adherence to the CBT manual and the quality of the CBT intervention. The assessment is undertaken by a CBT therapist independent of the study delivery team with feedback given as needed.

### Process evaluation

A process evaluation will be carried out as described by previous research [18].

The following outcomes assessed: The proportion of the population of the intended target population that actually participated in the intervention, the number of patients who completed the intervention, the mean number of CBT sessions and patient satisfaction of the intervention and patient information leaflets.

### Sample size

Complete outcome data is expected to be available on 112 patients per group (224 in total). This gives 80% power to detect a standardised difference of 0.375 (equivalent to a change in HADS-A of at least 1.5 when standard deviation is assumed at 4) assuming a type 1 error rate of 5%.

### Analysis

An “Intention to treat” analysis is planned. Descriptive statistics will be used for analysis. The sample size calculation assumes that groups will be compared using an independent *t*-test (the primary outcome variable being the HADS-A score at three months). For data collected at multiple points in time, a longitudinal analysis will be undertaken. This will involve comparing the way HAD scores change over time in the different trial arms using repeated measures analysis of variance such as a mixed effects or multilevel model (repeat assessments nested within patients). This will make use of all the observed data for a patient even if they missed one or more time points. Finally the effect of missing data on our results will be estimated.

### Health economic evaluation

An economic evaluation will be undertaken. Costs of respiratory admissions, cost of CBT intervention, cost of attending clinic, QALY’s (Quality of adjusted life year saved as per EQ5D) will be assessed.

### Trial steering committee

A trial steering committee has been formed to advise and monitor the study. The steering committee includes a patient and carer with COPD and a representative from the British Lung Foundation Charity. The committee has a number of experts and patient representatives including:

• Consultant Chest Physician (Chair).

• Representative for the British Lung Foundation and advisor for service users.

• A carer & service user.

• An expert in trial design.

• Respiratory Nurse Consultant & CBT therapist (Trial management/.Recruitment/Consent/Cognitive Behavioural Therapy & training respiratory nurses).

• A Chest Physician & academic researcher.

• Head of Nursing and Midwifery Research, an expert in research ethics and governance.

• A Clinical Psychologist will advise the project team particularly in respect of development of manualised training and provide clinical supervision.

• Medical statistician.

There will be overall oversight by a Trials Steering Committee (TSC) that will meet three times a year. As CBT is frequently undertaken within clinical care it is envisaged that this study poses minimum safety risks therefore a decision has been made not to have a separate study management group. However, recruitment, quality of data and safety of participants will be reviewed at each trial steering committee.

## Discussion

This study design has evolved through literature review, contact with published authors, a case series defining local COPD anxiety & depression rates [19] and a non-randomised case series of CBT in COPD [17]. Further study design refinement was through the National Institute of Health Research Design Service. This research builds upon and extends the prior work [15,16,20-24]. The study will address impact, feasibility and efficacy of teaching CBT skills to non-mental health professionals (such as respiratory nurses), to treat anxiety and depression in a clinical population of COPD patients. This is particularly important as there is a national shortage of CBT therapists. In routine clinical practice, CBT delivery by respiratory nurses could be the most realistic and potentially cost effective model to provide psychological care for patients with COPD. Respiratory nurses are front line staff who work with high volumes of COPD patients and will have the skill sets that may allow distinction between organic and psychological causes of breathlessness. The ability to integrate aspects of COPD care within one (familiar) setting such as a respiratory clinic or with familiar staff may help reduce barriers to engagement with the mental health aspects of COPD.

## Abbreviations

BMI: Body mass index; CAT: COPD assessment test; CBT: Cognitive behavioural therapy; CLRN: Clinical research network; COPD: Chronic obstructive pulmonary disease; EQ5D: Euroqol- 5 health state; FVC: Forced vital capacity; FEV1: Forced expiratory volume; QALY’s: Quality of adjusted life year saved; HADS: Hospital anxiety & depression scale; MRC: Medical research council; NHS: National health service; NIHR: National Institute of Health Research; TSC: Trial steering committee.

## Competing interests

The authors declare that they have no competing interests.

## Authors’ contributions

KH is investigator and wrote the manuscript and study protocol with input from the other authors. ADS/DCS/GB/CB/JN supervised the planning and progress of the project. All authors read, edited and approved the final manuscript.

## Pre-publication history

The pre-publication history for this paper can be accessed here:

http://www.biomedcentral.com/1471-2466/13/62/prepub

## References

[B1] National Institute of Clinical Excellence Chronic obstructive pulmonary disease: management of chronic obstructive pulmonary disease in adults in primary and secondary care 2010 London: National Clinical Guideline

[B2] Bourke SB Burns GP Lecture notes: respiratory medicine 2011 8 Wiley-Blackwell Publications

[B3] Ng TP Niti M Tan WC Cao Z Ong KC Eng P Depressive symptoms and chronic obstructive pulmonary disease: effect on mortality, hospital readmission, symptom burden, functional status, and quality of life Arch Intern Med 2007 167 1 60 67 10.1001/archinte.167.1.60 17210879

[B4] Department of Health Outcome strategy for chronic obstructive pulmonary disease and asthma in England 2011 https://www.gov.uk/government/publications/an-outcomes-strategy-for-people-with-chronic-obstructive-pulmonary-disease-copd-and-asthma-in-england

[B5] British Lung Foundation Lost in translation: bridging the communication gap in COPD 2006 London: BLF

[B6] Naylor C Parsonage M McDaid D Knapp M Fossey M Galea A Long-term conditions and mental health. The cost of co-morbidities 2012 The King’s Fund and Centre for Mental Health http://www.kingsfund.org.uk/sites/files/kf/field/field_publication_file/long-term-conditions-mental-health-cost-comorbidities-naylor-feb12.pdf

[B7] Yohannes AM Baldwin RC Connolly MJ Depression and anxiety in elderly patients with chronic obstructive pulmonary disease Age Ageing 2006 35 5 457 459 10.1093/ageing/afl011 16638758

[B8] David L Using CBT in general practice. The 10 minute consultation 2006 Bloxham, Oxfordshire, UK: Scion Publishing Limit

[B9] Giardino ND Curtis JL Abelson JL King AP Pamp B Liberzon I Martinez FJ The impact of panic disorder on interoception and dyspnea reports in chronic obstructive pulmonary disease Biol Psychol 2010 84 1 142 146 10.1016/j.biopsycho.2010.02.007 20176074

[B10] Yohannes AM Baldwin RC Connolly MJ Depression and anxiety in elderly outpatients with chronic obstructive pulmonary disease: prevalence, and validation of the BASDEC screening questionnaire Int J Geriatr Psychiatry 2000 15 12 1090 1096 10.1002/1099-1166(200012)15:12<1090::AID-GPS249>3.0.CO;2-L 11180464

[B11] Brenes GA Anxiety and chronic obstructive pulmonary disease: prevalence, impact, and treatment Psychosom Med 2003 65 6 963 970 10.1097/01.PSY.0000097339.75789.81 14645773

[B12] Livermore N Sharpe L McKenzie D 'Panic attacks and panic disorder in chronic obstructive pulmonary disease: a cognitive behavioral perspective’ Respir Med 2010 104 9 1246 1253 10.1016/j.rmed.2010.04.011 20457513

[B13] Livermore N Sharpe L McKenzie D Prevention of panic attacks and panic disorder in COPD Eur Respir J 2010 35 3 557 563 10.1183/09031936.00060309 19741029

[B14] Baraniak A Sheffield D The efficacy of psychologically based interventions to improve anxiety, depression and quality of life in COPD: a systematic review and meta-analysis Patient Educ Couns 2011 83 1 29 36 10.1016/j.pec.2010.04.010 20447795

[B15] National Institute of Clinical Excellence Generalised anxiety disorder in adults, The NICE guideline on management in primary, secondary and community care. National collaborating Centre for Mental Health, The British Psychological Society & The Royal College of Psychiatrist 2011 http://publications.nice.org.uk/generalised-anxiety-disorder-and-panic-disorder-with-or-without-agoraphobia-in-adults-cg113

[B16] National Institute of Clinical Excellence Depression in adults with a chronic physical health problem. Treatment and management. CG 91 2010 National Collaborating Centre for Mental Health http://publications.nice.org.uk/depression-in-adults-with-a-chronic-physical-health-problem-cg91

[B17] Heslop K De Soyza A Baker CR Stenton C Burns GP Using individualised cognitive behavioural therapy as a treatment for people with COPD Nurs Times 2009 105 14 14 17 19449602

[B18] Lamers F Jonkers CCM Bosma H Diederiks JPM van Eijk JTM Effectiveness and cost-effectiveness of a minimal psychological intervention to reduce non-severe depression in chronically ill edlerly patients: the design of a randomised controlled trial BMC Public Health 2006 http://www.biomedcentral.com/1471-2458/6/161/prepub 10.1186/1471-2458-6-161 PMC1555592 16790039

[B19] Yates B Walker E Burns GPB Depression and COPD Am J Respir Crit Care 2007 175 A643

[B20] Kunik ME Braun U Stanley MA Wristers K Molinari V Stoebner D Orengo CA 'One session of cognitive behavioural therapy for elderly patients with chronic obstructive pulmonary disease Psycholo Med 2001 31 4 717 723 10.1017/s0033291701003890 11352373

[B21] Kunik ME Veazey C Cully JA Souchek J Graham DP Hopko D Carter R Sharafkhaneh A Goepfert EJ Wray N Stanley MA 'COPD education and cognitive behavioural therapy group treatment for clinically significant symptoms of depression and anxiety in COPD patients: a randomized controlled trial’ Psychol Med 2008 38 3 385 396 17922939 10.1017/S0033291707001687

[B22] Lamers F Jonkers CC Bosma H Kempen GI Meijer JA Penninx BW Knottnerus JA van Eijk JT A minimal psychological intervention in chronically ill elderly patients with depression: a randomized trial Psychother Psychosom 2010 79 4 217 226 10.1159/000313690 20424499

[B23] Howard C Dupont S Haselden B Lynch J Wills P The effectiveness of a group cognitive-behavioural breathlessness intervention on health status, mood and hospital admissions in elderly patients with chronic obstructive pulmonary disease Psychol Health Med 2010 15 4 371 385 10.1080/13548506.2010.482142 20677076

[B24] Hynninen MJ Bjerke N Pallesen S Bakke PS Nordhus IH A randomized controlled trial of cognitive behavioral therapy for anxiety and depression in COPD Respir Med 2010 104 7 986 994 10.1016/j.rmed.2010.02.020 20346640

